# Noninvasive spatiotemporal imaging of neural transmission in the subcortical visual pathway

**DOI:** 10.1038/s41598-017-04700-x

**Published:** 2017-06-30

**Authors:** Fumiaki Yoshida, Masayuki Hirata, Ayako Onodera, Tetsu Goto, Hisato Sugata, Shiro Yorifuji

**Affiliations:** 1 Endowed Research Department of Clinical Neuroengineering, Global Center for Medical Engineering and Informatics, Osaka University, Suita, Osaka Japan; 2 Department of Neurosurgery, Osaka University Medical School, Suita, Osaka Japan; 3 Center for Information and Neural Networks (CiNet), National Institute of Information and Communications Technology, and Osaka University, Suita, Osaka Japan; 4 Division of Functional Diagnostic Science, Osaka University Graduate School of Medicine, Suita, Osaka Japan

## Abstract

Spatiotemporal signal transmission in the human subcortical visual pathway has not been directly demonstrated to date. To delineate this signal transmission noninvasively, we investigated the early latency components between 45 ms (P45m) and 75 ms (N75m) of visually-evoked neuromagnetic fields (VEFs). Four healthy volunteers participated in this study. Hemi-visual field light flash stimuli were delivered a total of 1200 times. Neuromagnetic responses were measured with a 160-channel whole-head gradiometer. In three participants, averaged waveforms indicated a subtle but distinct component that peaked with a very early latency at 44.7 ± 2.1 ms with an initial rise latency of 36.8 ± 3.1 ms, followed by a typical prominent cortical component at 75 ms. The moving equivalent current dipoles continuously estimated from P45m to N75m were first localized in the vicinity of the contralateral lateral geniculate body, then rapidly propagated along the optic radiation and finally terminated in the contralateral calcarine fissure. This result indicates that the source of P45m is the lateral geniculate body and that the early latency components P45m–N75m of the VEFs reflect neural transmission in the optic radiation. This is the first report to noninvasively demonstrate the neurophysiological transmission of visual information through the optic radiation.

## Introduction

Visual evoked responses are a useful tool for evaluating visual function and are used to detect visual disturbances in clinical practice. Visual evoked responses that are detected using magnetoencephalography (MEG) are called visual evoked magnetic fields (VEFs), which typically have several peaks at 75 ms, 100 ms and 145 ms after the presentation of visual stimuli. These peaks are termed N75m, P100m and N145m, respectively, and all of these VEFs are thought to originate within the primary visual cortex^1–3^. Visual evoked potentials (VEPs) that are detected using electroencephalography (EEG) exhibit a paradoxical lateralization in which the maximal response occurs at the scalp electrodes that are situated over the hemisphere ‘ipsilateral’ to the field that was stimulated^4, 5^. By contrast, VEFs clearly distinguish bilateral occipital responses from each other using left- or right-hemifield stimuli^6–9^. Therefore, a unilateral abnormality in the visual cortex or an interhemispheric difference between the left and right cortices can be properly detected^10^. Any abnormal visual function can be quantitatively evaluated by the latency delay with or without amplitude attenuation^11, 12^. Therefore, the spatial localization of visual cortical function can be more precisely assessed with VEFs than with VEPs. Furthermore, VEFs have early components in response to flash stimuli with a latency ranging from 42 to 100 ms^7, 8, 13–19^. The origin of the early latency components of VEFs remains unclear. Furthermore, to date, the spatiotemporal neural transmission in the human subcortical visual pathway has not been directly demonstrated. The optic tract transmits a sequence of signals from the retina to the visual cortices. The functional damage in this pathway due to brain injuries, strokes and brain tumours may result in a disturbance in visual acuity or visual fields. Therefore, it is important to noninvasively evaluate the functional visual pathway.

MEG is a noninvasive method that has a high spatiotemporal resolution^20^ and is, thus, suitable for accurately detecting the localization of these transient responses in humans. Kimura *et al*.^21^ previously visualized impulse propagation along the thalamo-cortical fibre tracts in the white matter with MEG and sufficient periods of somatosensory electrical stimulation. However, functional visualization of the visual pathway has not been reported previously. Here, we hypothesized that the spatiotemporal distribution of the subtle current sources in the subcortical visual pathway that are activated by visual stimulation can also be detected by MEG if the type, times and frequency of the visual stimuli are appropriate. To visually delineate the spatiotemporal signal transmission in the human subcortical visual pathway noninvasively, we investigated the early latency components of neuromagnetic visual responses. Although the number of participants is limited, this report is the first to demonstrate the functional visualization of the visual pathway.

## Results

### MEG data Analyses

**Figure 1:**
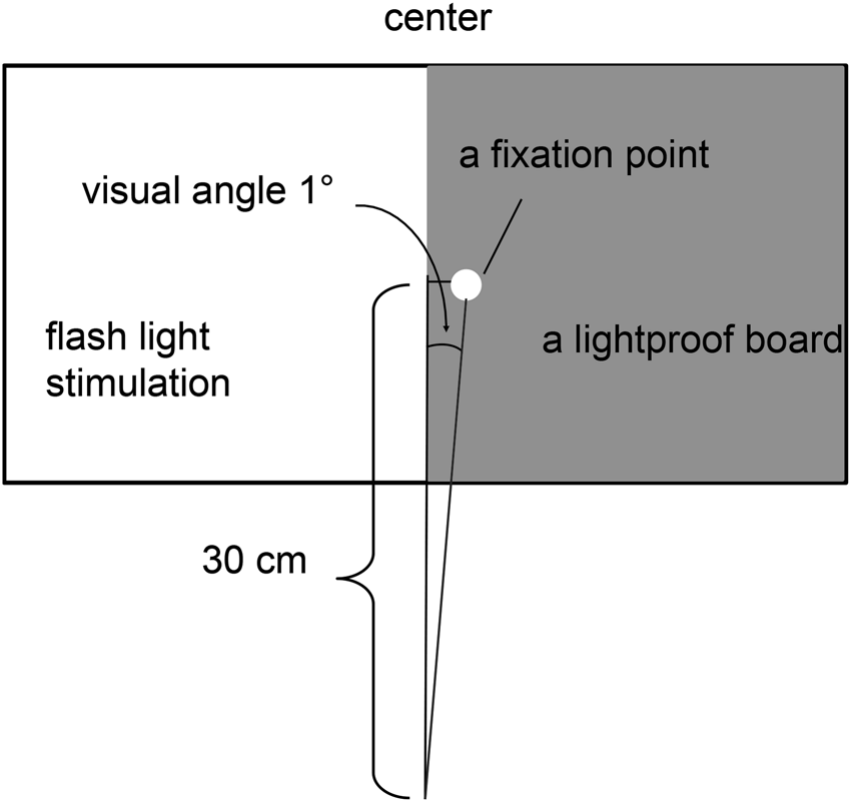
Visual stimulation. Hemi-visual field light flash stimuli were delivered from outside of the shielded room using reflecting mirrors. In total, 1200 visual stimuli were delivered at random intervals of 2000 ± 200 ms.

**Figure 2:**
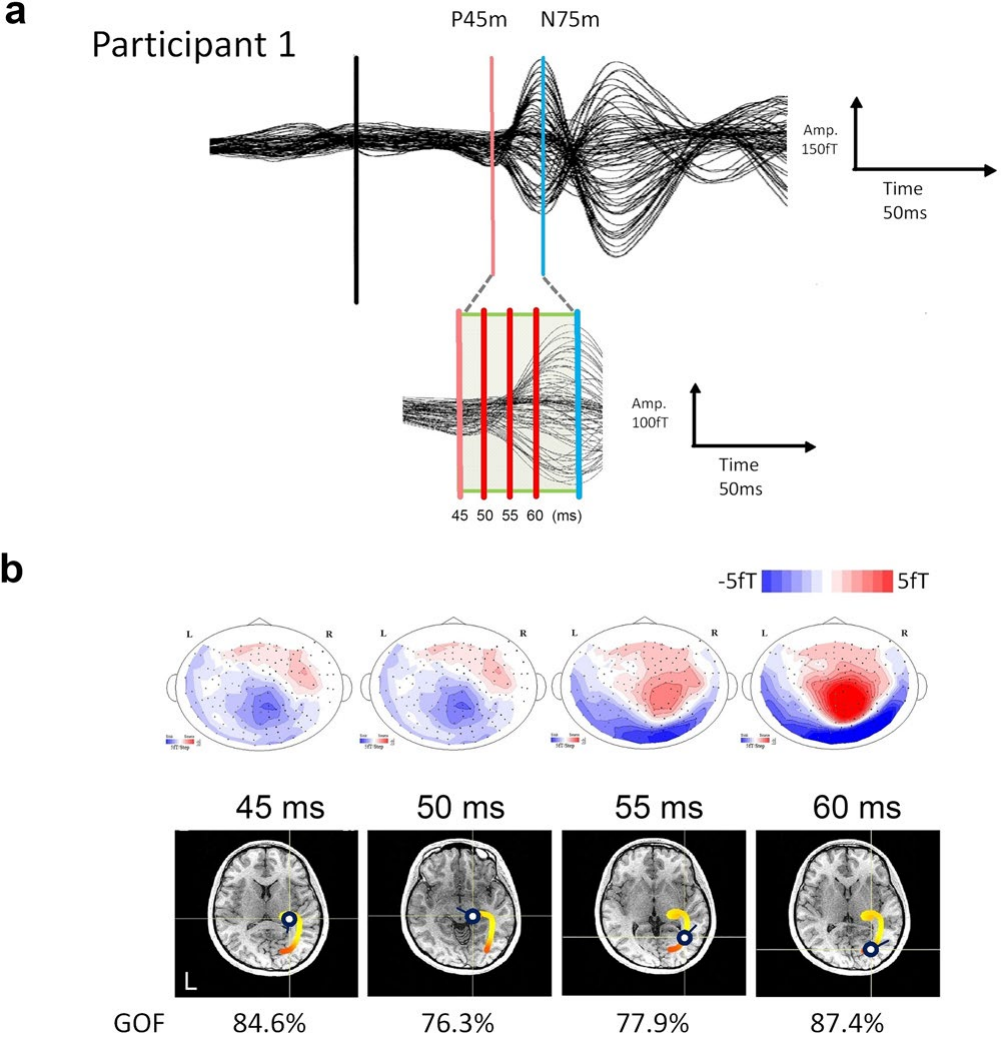
Representative visually evoked neuromagnetic fields following hemi-visual field light flash stimuli in a healthy volunteer. (**a**) Superimposed waveforms recorded from 80 locations of the right hemisphere depict the P45m and N75m deflections. Four points in the time sequence from 45 ms to 60 ms were analysed. (**b**) **top**, Estimated ECDs superimposed onto the horizontal plane of the participant’s MRI scan. Changes in the locations of the ECDs were calculated successively in 0.1-ms steps between 45 ms and 60 ms after the stimulus presentation. The C-shaped coloured curve shows the sequential changes in the ECD location from the LGN (light yellow) to the visual cortex (red). (**b**) **bottom**, Top view of the isocontour field distributions of the magnetic field over the head surface at 4 time points; red contours, magnetic source; blue contours, sink with a contour step of 5 fT for the latency of 45 ms to 60 ms. Note that the long distance between the maximum source and minimum sink and the wide intervals between any adjacent contour lines for the source or sink (45 ms and 50 ms) indicate deep current sources; by contrast, the short distance and narrow intervals between the adjacent contour lines (55 ms and 60 ms) indicate superficial current sources.

In total, 1200 hemi-visual field light flash stimuli were delivered at random intervals (Fig. 1), and the VEF responses were measured. All participants exhibited a clear typical cortical component at 75 ms (N75m), and the corresponding equivalent current dipoles (ECDs) were localized near the contralateral calcarine fissure in the striate cortex (primary visual cortex, V1). The ECD of N75m pointed outwards in all participants. In three of the four participants, the averaged waveforms indicated a subtle but distinct very early latency component with a peak at 44.7 ± 2.1 ms (P45m, initial rise latency: 36.8 ± 3.1 ms). The mean maximum signal strength of N45m was 24.9 femtotesla (fT). Figure 2a illustrates the VEF results following the left flash stimulation that were obtained from a representative participant. Figure 2b (top) shows the isocontour field maps of the magnetic fields between the P45m and N75m components. The long distance between the maximum flux-out and minimum flux-in over the head surface for the N45m component and the wide intervals between any adjacent contour lines for the flux-out or flux-in (Fig. 2b top 45 ms, 50 ms) reflect deep current sources for N45m. These findings present a striking contrast to those of the N75m component, which is located superficially in the visual cortex, displays a magnetic field pattern with a short distance between the maximum flux-out and minimum flux-in and has narrow intervals between the adjacent contour lines (Fig. 2B top 55 ms, 60 ms). We estimated the location and orientation of the successive ECDs from the N45m onset to the N75m peak. Figure 2b (bottom) illustrates the changes in the location of the ECDs for the N45m–N75m continuum using a colour gradation from light yellow to red that corresponds to the time courses shown in Fig. 2a. The moving ECDs continuously estimated from P45m to N75m were localized in a C-shaped coloured curve in the axial plane of the MRI (Fig. 2b bottom), which, at first, was in the vicinity of the contralateral lateral geniculate body (light yellow), then rapidly moved along the optic radiation, finally reached the contralateral calcarine fissure at approximately 60 ms after the visual stimuli and remained there until 75 ms (red).

**Figure 3:**
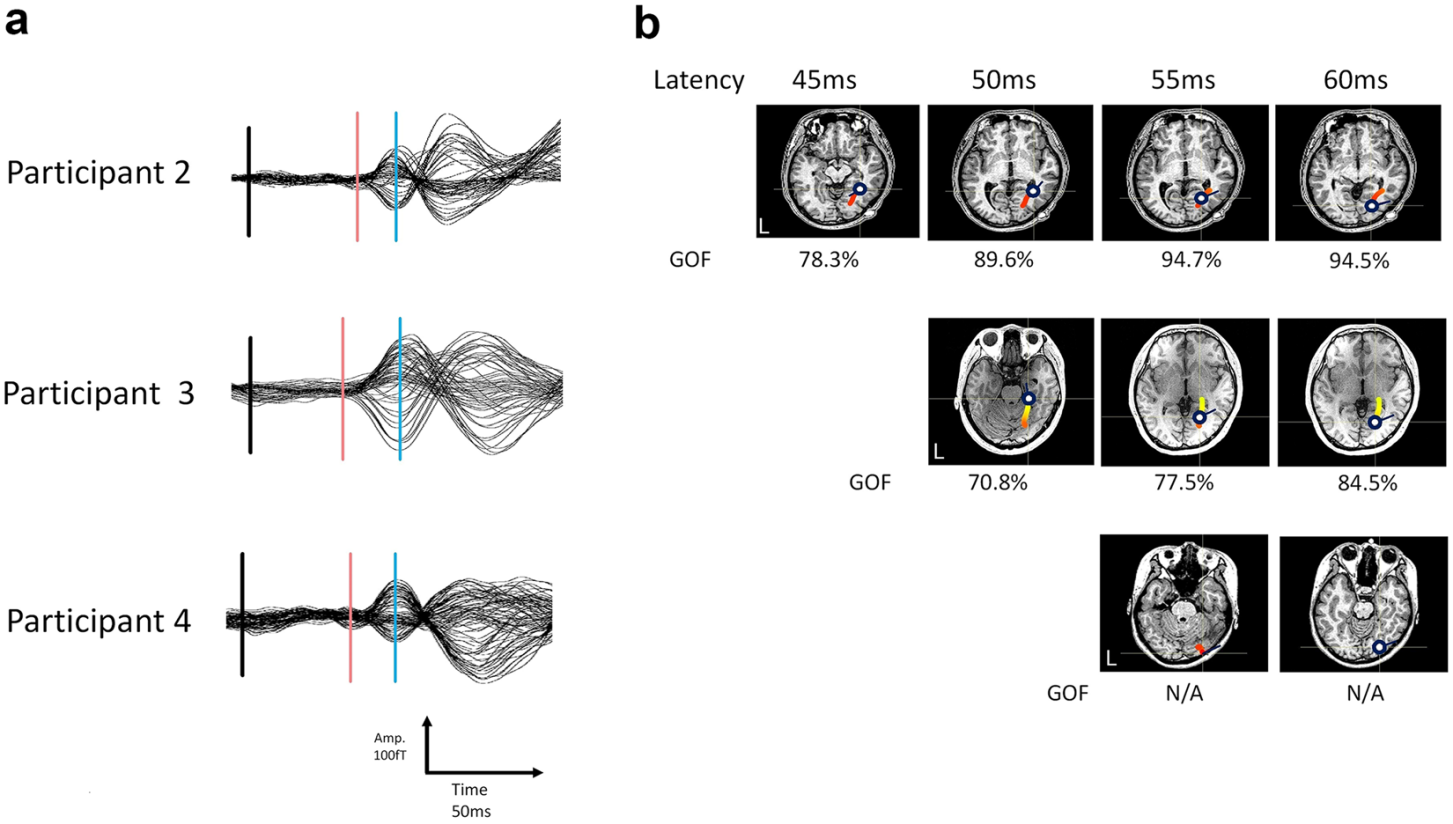
Averaged waveforms and the moving ECDs at 45–60 ms for the other three participants. (**a**) Averaged waveforms. Red lines indicate P45m, and blue lines indicate N75m as described in Fig. 2. Participant 3 did not clearly exhibit a P45m component, and we analysed the signals between 50 ms and 60 ms. (**b**) Estimated ECDs superimposed onto the horizontal plane of the MRI scan of each participant. Changes in the locations of the ECDs were calculated successively in 0.1-ms steps from 45 ms (participant 2), 50 ms (participant 3), and 55 ms (participant 3) to 60 ms after the presentation of the visual stimuli. Note that the GOF values were obtained from two participants and were between 70 and 95%. An axial coloured bar with a colour gradation from light yellow to red corresponds to the time course of the 45- to 60-ms continuum. In participants 2 and 3, the moving ECDs that were continuously estimated were localized first in the vicinity of the LGN and then moved along the optic radiation to the contralateral visual cortex.

Figure 3 shows the results of the ECD analysis of the other three participants. The changes in the locations of the ECDs were calculated successively at 0.1-ms steps from 45 ms (participant 2), 50 ms (participant 3), or 55 ms (participant 4) to 60 ms after the stimulus onset. P45m was not detected in participant 3; therefore, the ECD analysis was performed after 50 ms. In participant 4, the ECDs were not estimated at 45 ms and 50 ms but were estimated at 55 ms and 60 ms and were localized in the primary visual cortex. The goodness-of-fit (GOF) values of all ECDs were in the range of 70.8–94.7%.

## Discussion

In the present study, we hypothesized that the spatiotemporal distribution of the subtle current sources in the subcortical visual pathway activated by visual stimulation could be noninvasively detected by MEG if the type, times and frequency of the visual stimuli were appropriate (Supplementary Information). Using moving ECDs, we investigated the early latency components of the neuromagnetic visual responses evoked by 1200 hemi-visual field light flash stimuli from P45m to N75m. Thus, the ECDs from P45m to N75m noninvasively delineated the neural transmission of the visual information through the subcortical visual pathway. In this section, we discuss the validity and significance of the findings of the present study with reference to the previous literature.

First, the trajectory of the moving ECDs between P45m and N75m corresponded well with the anatomical subcortical visual pathway. The anatomical subcortical visual pathway originates in the lateral geniculate body via Meyer’s loop and the optic radiation and terminates in the occipital cortex near the calcarine fissure^22^. In the present study, the moving ECDs were localized first in the vicinity of the contralateral lateral geniculate body, then rapidly propagated along Meyer’s loop and the optic radiation, and finally terminated in the contralateral calcarine fissure. The trajectories were identical to the anatomical visual pathway. MR diffusion anisotrophy also offered noninvasive visualization of the anatomical subcortical visual pathway^23^. However, to date, there are no established methods that allow the visualization of the dynamic process of the neurophysiological transmission of visual information.

Regarding the thalamocortical somatosensory pathway, several studies have succeeded in noninvasively imaging the neural transmission of somatosensory information^21, 24–26^. Kimura *et al*. explained how they detected the action potentials that propagated along the subcortical fibres in the deep brain, which had been considered undetectable by MEG measurement. Although it has been thought that for deep sources the magnetic fields produced by the primary intracellular currents are largely cancelled out by the fields due to secondary extracellular currents, this study suggests the possibility that these primary currents can be selectively detected by MEG^21^.

In the present study, P45m was obtained in three of the four participants using hemi-flash stimulation, and the ECD was estimated to be in the vicinity of the contralateral lateral geniculate body. In our study, the initial rise in the latency of the P45m peak was 36.8 ± 3.1 ms. This result is comparable to that previously reported (36.6 ± 0.13 ms) using macaque lateral geniculate nucleus (LGN) recordings by Munsell *et al*.^27^. Poghosyan *et al*. reported data from humans showing that the initial feed-forward response in the V1 begins at 55 ms and peaks at 70 ms after the presentation of visual stimuli. Our results indicating that the moving ECDs departed the LGN at 50 ms, propagated in the optic radiation at 55 ms, reached the V1 at 60 ms, and peaked at 70 ms are consistent with these previous findings.

In addition, the N75m dipole pointed outwards in the contralateral primary visual cortex of all participants, which is consistent with previous reports^1–3^. This fact indicated that our recording and analyses were properly performed. Yoneda *et al*.^28^ proposed that the origin of N75m was V1. However, VEFs are significantly influenced by the stimulation parameters and recording conditions. A low stimulation frequency and relatively weak flashes can emphasize deep brain activities rather than cortical activities. The experiments by Yoneda’s group were performed with a 37-channel MEG system, 500 stimuli, and a sampling rate of 2 kH. In contrast to their experimental settings, our experiment was carried out with a 160-channel system, 1200 stimuli and a 10 kH sampling rate. These enhanced experimental settings might have enabled the successful detection of subcortical activities.

Our study is the first to demonstrate the visualization of neural transduction in the visual pathways. We suggest that MEG may be a useful tool to depict the processing of white matter information and cortical information. This method can be applied to other white matter tracts, such as the motor pathway, if we employ adequate measuring parameters. In addition, MEG can be used to detect malfunctions in the white matter fibre pathways.

We emphasize that we aimed to provide ‘proof of feasibility’ in a moderately small number of subjects. The accuracy of the result needs to be confirmed in a larger and more detailed study. Moreover, our hypothesis that the results are due to transduction rather than to other factors remains to be definitively established.

In conclusion, we demonstrated that the source of P45m is the lateral geniculate body and that the early latency components P45m–N75m of the visually evoked neuromagnetic fields reflect neural transmission in the optic radiation. This is the first report to noninvasively demonstrate the neurophysiological transmission of visual information through the optic radiation.

## Methods

### Participants

Four healthy subjects (2 females, 2 males; age: 24.3 ± 2.1 years, range: 22–27 years) participated in this study. None of the participants had a history of neurological or psychiatric disease, abnormal visual fields or visual disturbances. All participants were normally sighted or had corrected-to-normal vision. In accordance with the Declaration of Helsinki, we explained the purpose and possible consequences of this study to all participants and obtained informed consent prior to their participation in the study. All methods were performed in accordance with the relevant guidelines and regulations. This study was approved by the ethical review board of Osaka University Hospital.

### Visual stimulation

Hemi-visual field light flash stimuli of 20 J (1460 cd as a point source) were delivered to each participant with a xenon light stimulator (LS-703A, Nihon-koden, Tokyo, Japan) from the outside of a magnetically shielded room using reflecting mirrors and a rear projection screen located at a distance of 30 cm from the eyes (Fig. 1). In total, 1200 visual stimuli were delivered at random intervals of 2000 ± 200 ms, with an approximate duration of 7 ms. The eye fixation point was also displayed on the screen, which was a white circle located at the right side by 1 degree in the visual angle from the midline. The right side from the midline of the screen was covered by a light-proof black board to confirm the left hemi-visual field stimulation. The fixation point subtended 1 degree in radius. The flash and room luminance were 2.0 cd/m^2^ and 0.7 cd/m^2^, respectively, with a contrast level of 49%. The participants were dark-adapted for 15 minutes before the experiment.

### MEG data acquisition

MEG signals were recorded at a sampling rate of 10 kHz using a 160-sensor whole-head type axial gradiometer system (MEG vision PQ1160C, Yokogawa Electric, Tokyo, Japan). The continuously recorded data were filtered online with a 0.3−200-Hz band-pass filter and a 60-Hz notch filter to eliminate the AC line noise. The data were separated into trials with durations of 600 ms (from −200 ms to 400 ms) based on the previous stimulus. The baseline of each trial was defined according to the 200 ms pre-stimulus interval. Finally, 1200 responses were averaged online. The method used for the stimulus was selected following a process to identify the most appropriate stimulus (Supplementary Information).

### Magnetic resonance imaging

Using a 3.0 T magnetic resonance imaging (MRI) system (SIGMA 3.0 T, GE Medical Systems, Waukesha, WI, USA), all participants underwent T1-weighted MRI. The MRI consisted of 216 sequential slices with a 0.9-mm thickness and a resolution of 512 × 512 points in a 240 × 240 mm field of view. To define the head shape in three dimensions for the subsequent computations of the source localization, we traced the area of the scalp using a sensor position indicator with standard fiducial points, including the left and right preauricular points and nasion (orbito-meatal line). After reconstructing a three-dimensional MRI scan, the best-fit sphere was chosen for each participant’s head.

### Analyses

A single ECD was continuously estimated with a 0.1-ms step from P45m to N75m using the data from 80 channels that were selected from 160 channels such that the selected channels covered both the inflow and outflow of magnetic flux. We accepted ECDs with a GOF value greater than 70% and superimposed the accepted ECDs onto each individual participant’s MRI scan to confirm the anatomical locations.

## Electronic supplementary material

Supplementary Information
