# Supplementary material for: Noninvasive spatiotemporal imaging of neural transmission in the subcortical visual pathway
Source: Sci Rep. 2017 Jun 30;7:4424. doi: 10.1038/s41598-017-04700-x (PMC5493626; doi:10.1038/s41598-017-04700-x)
Supplement: Supplementary file 1 — Supplementary Information [file 41598_2017_4700_MOESM1_ESM.pdf]

Noninvasive spatiotemporal imaging of neural transmission in the subcortical visual pathway

Fumiaki Yoshida, Masayuki Hirata, Ayako Onodera, Tetsu Goto, Hisato Sugata, Shiro Yorifuji

Supplementary Information

We performed preliminary experiments to identify the most appropriate visual stimuli for the detection of P45m. First, we tested the following two types of visual stimuli: flash stimuli and pattern reversal stimuli. Then, we tested the following stimulus times: 1200 times and 2400 times. Finally, we tested the following three different frequencies: 2 Hz, 1 Hz and 0.5 Hz.

1. Stimulus type

First, we compared flash stimuli and pattern reversal stimuli in a single participant. The pattern reversal stimuli evoked a more prominent N75m response but also caused a larger slow component even at 0 ms compared with that using the flash stimuli (Supplementary figure 1). This slow component persisted immediately before the N75m component. The ECD of this component at 0 ms was localized in the occipital area.

The pattern reversal stimuli consist of a combination of black and white areas. The spatial pattern of the pattern reversal stimuli is more complicated than that of the flash stimuli. This complex pattern most likely caused late evoked components in the occipital area, which might persist not only at 0 ms but also until immediately before the next N75m, implying an overlapping effect. However, the flash stimuli did not cause a component throughout the pre-stimulus period from -50 ms to the P45m. Therefore, we determined that the flash stimuli were more appropriate for measuring P45m than the pattern reversal stimuli.

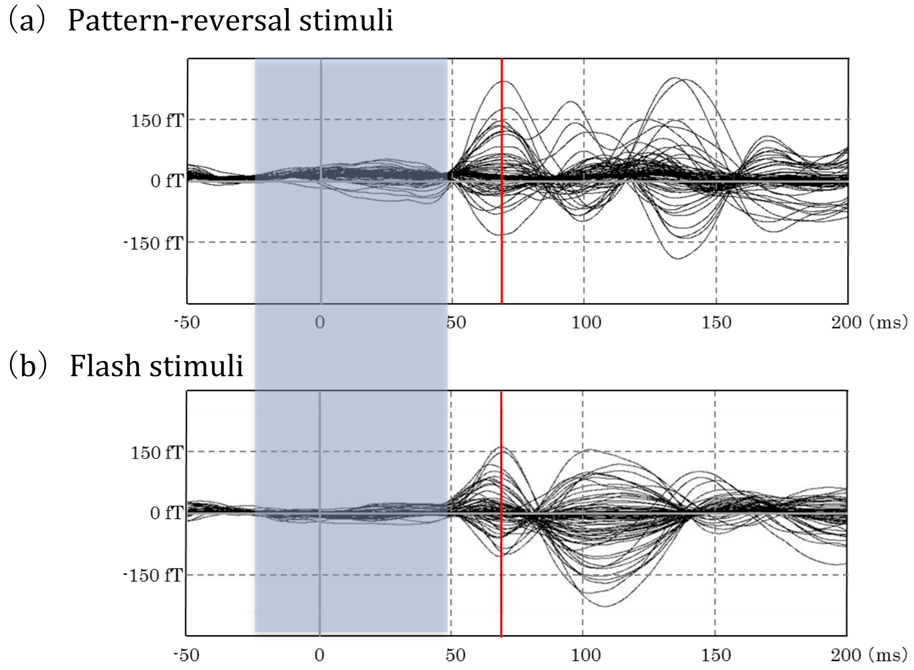

Supplementary Figure 1. Comparison of visual evoked fields between the pattern-reversal stimuli and flash stimuli. Vertical axis indicates the response amplitude, and horizontal axis indicates the latency. Red line indicates N75m. Both the pattern reversal （a） and flash stimuli （b） produce N75m. The flash stimuli show better baseline uniformity.

2. The number of stimulus presentations

Second, we compared 1,200 and 2,400 stimulations. For P45m, the peak signal-to-noise ratios of the amplitudes of the VEFs were 1.4 when the stimuli were presented 1200 times and 1.9 when the stimuli were presented 2,400 times (Supplementary Table 1). For P75m, the peak signal-to-noise ratio was 6.3 when the stimuli were presented 1200 times and 12.5 when the stimuli were presented 2,400 times.

Supplementary table 1. Comparison of the amplitudes of P45m and N75m between 1,200 and 2,400 stimulus presentations

	Number of presentations	Peak signal amplitude (fT)	Noise level(fT)	Signal /noise ratio
P45m	1200	35.8	25.3	1.4
	2400	23.5	12.6	1.9
N75m	1200	159.1	25.3	6.3
	2400	157.0	12.6	12.5

In reality, a large number of stimulations may detect even subtle responses upon averaging. However, 2400 stimulations at a sampling rate of 0.5 Hz requires up to 80 minutes. Presenting flash stimuli for 80 minutes does not only cause fatigue but is also very uncomfortable. Therefore, although our preliminary comparison indicated that 2400 stimulations showed clearer response compared to 1200 stimulations, we determined that 1,200 stimulations were more feasible.

3. Stimulus frequency

We also examined several patterns of stimulation frequency to clarify the best frequency for our purpose. The frequencies we tested were 2 Hz, 1 Hz and 0.5 Hz. Overall, N75m was observed in all conditions. P45m was observed only under the 1 Hz and 0.5 Hz conditions (Supplementary figure 2). We calculated the signal-to-noise ratio using noise at time 0 as the baseline (Supplementary table 2). Compared with the noise amplitude at 0 ms with 2 Hz (37.5 fT), the ratio was small under the 1 Hz (30.8 fT) and 0.5 Hz (19.6 fT) conditions. These phenomena were assumed to occur because of a long latency response effect on the next response when we employed high-frequency stimuli. We decided to use the 0.5 Hz stimuli in our experiment.

（a） 2 Hz stimuli

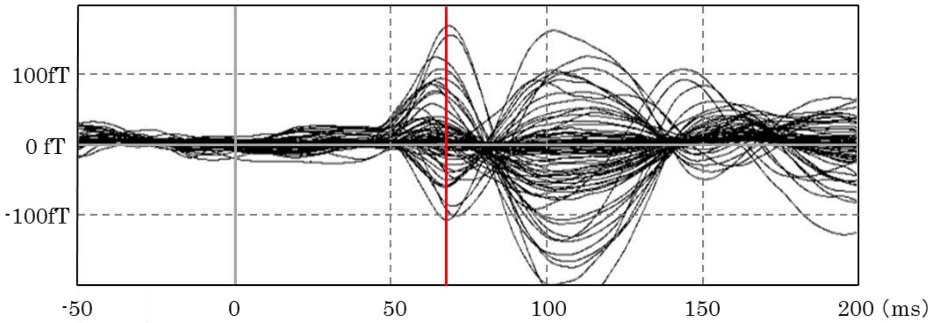

（b） 1 Hz stimuli

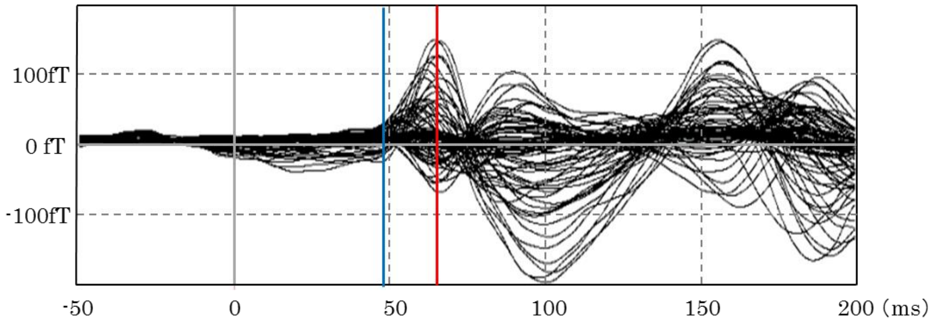

（c） 0.5 Hz stimuli

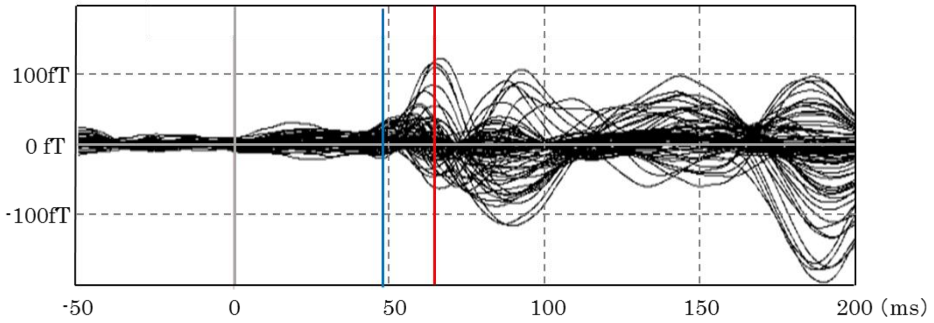

Supplementary Figure 2. Comparison of visual evoked fields with 2 Hz, 1 Hz and 0.5 Hz flash stimuli. Vertical axis indicates the response amplitude. Horizontal axis indicates latency. The red line indicates N75m, and the blue line indicates P45m. (a） 2 Hz, (b） 1 Hz, (c） 0.5 Hz. Although N75m was observed under all conditions, P45m was observed only under the 1 Hz and 0.5 Hz conditions.

Supplementary Table 2. Comparison of amplitude between stimulus frequencies of 2 Hz, 1 Hz and 0.5 Hz

	Frequency (Hz)	Amplitude (fT)	Noise level(fT)	Signal /noise ratio
P45m	2.0	N/D	37.5	N/D
	1.0	50.8	30.9	1.7
	0.5	49.9	19.6	2.5
N75m	2.0	318.9	37.5	8.5
	1.0	212.9	30.9	6.8
	0.5	179.5	19.6	9.1

N/D: Not Detected
